# Current News and Opinion

**Published:** 1882-11

**Authors:** 


### CURRENT NEWS AND OPINION.

DR. EDWARD C. SEGUIN, a physician of considerable reputation as a necrologist in our city, on returning to his home from his daily practice on the 1st inst., found that his wife had killed their three children and committed suicide. She evidently did this rash act in a temporary state of insanity, as she was up to this time a model wife and devoted mother. Dr. Seguin has our warmest sympathy as well as that of the medical fraternity of New York. Several of our medical societies have passed appropriate resolutions expressing sympathy with the Doctor in his affliction. He has sailed for Europe with the intention of being absent a long and indefinite period.

AN EPIDEMIC OF TYPHOID FEVER rages at Providence, R. I.

THE YELLOW FEVER has about subsided at Pensacola, Fla. The total number of cases there during the epidemic were 2,330 with only 191 deaths.

SIR THOMAS WATSON, ninety-one years old, is now prostrated under a stroke of paralysis and is not expected to recover. He retains his mental faculties, and said when stricken: " This is the beginning of the end."

THE NEW YORK MEDICAL JOURNAL AND OBSTETRICAL REVIEW, a monthly, will, after the 1st of January, 1883, be published weekly.

TOY PISTOLS are legally abolished in Vermont.

TWO NEW POST-GRADUATE MEDICAL SCHOOLS opened their first sessions in this city on the 6th and 7th insts. The schools are known as the New York Post Graduate Medical School, and the New York Polyclinic. As our city grows why not our scientific institutions? New York is *the* city for clinical material and professional ability. Hitherto the profusion of this clinical supply has not been utilized. The Post-Graduate school gives didactic and clinical instruction. The Polyclinic is only intended for clinical teaching.

THE SIMS' RECEPTION, in honor of Prof. S. D. Gross, of Phila., at the Hotel Brunswick, was a brilliant gathering of the sons of Esculapiaus. They represented New York, Brooklyn, Philadelphia, Baltimore, Washington, Pittsburgh, Chicago, Boston, and other cities, numbering about four hundred, who came to do honor to two of America's most honored medical men—Sims and Gross. The hosts, Dr J. Marion Sims, and his son, Dr. Harry Marion Sims, entertained their guests in a manner becoming gentlemen with their reputation.

